# Supplementary material for: National Early Warning Score 2 (NEWS2) as a prognostic tool for adult patients in emergency department: A retrospective observational study
Source: PLoS One. 2025 Jun 16;20(6):e0326058. doi: 10.1371/journal.pone.0326058 (PMC12169516; doi:10.1371/journal.pone.0326058)
Supplement: S1 Table — (DOCX) [file pone.0326058.s001.docx]

**Supporting Information File 1**

1. **Sample size calculation for all 3 clinical outcomes**

1. NEWS2 score ≥5 in predicting critical care unit admission and in-hospital mortality for adult patients

Based on a study done by Thoren et al ([1](#_ENREF_1)), sensitivity and specificity for critical care unit admission and mortality prediction were 91.8% and 15.8% respectively.

With a prevalence of 85% of adult patients in ED HCTM (P = 0.85), z = 1.96 (CI = 95%) and w = 0.05, sample sizes for sensitivity N (Sn) and specificity N (Sp) are calculated as below:

| Sensitivity N (Sn) | Specificity N (Sp) |
| --- | --- |
| TP + FN = z^2^ x Sn (1-Sn)  w^2^    = 1.96^2^ x 0.918 (1 - 0.918)  0.05^2^    = 3.842 x 0.0753  0.0025  = 115.72    N (Sn) = TP + FN  P  = 115.72 = 136  0.85 | FP + TN = z^2^ x Sp (1-Sp)  w^2^    = 1.96^2^ x 0.158 (1 – 0.158)  0.05^2^    = 3.842 x 0.1330  0.0025  = 204.39    N (Sp) = FP + TN  (1-P)  = 204.39 = 1362  (1-0.85) |

2. NEWS2 score ≥5 in predicting critical care unit admission and in-hospital mortality for adult sepsis patients

Based on a study done by ([2](#_ENREF_2)), sensitivity and specificity for critical care unit admission and mortality prediction were 84% and 37% respectively.

With a prevalence of 13% of adult sepsis patients in ED HCTM (P = 0.13), z = 1.96 (CI = 95%) and w = 0.05, sample sizes for sensitivity N(Sn) and specificity N(Sp) are calculated as below:

| Sensitivity N (Sn) | Specificity N (Sp) |
| --- | --- |
| TP + FN = z^2^ x Sn (1-Sn)  w^2^    = 1.96^2^ x 0.84 (1 - 0.84)  0.05^2^    = 3.842 x 0.1344  0.0025  = 206.54    N (Sn) = TP + FN  P  = 206.55 = 1589  0.13 | FP + TN = z^2^ x Sp (1-Sp)  w^2^    = 1.96^2^ x 0.37 (1 – 0.37)  0.05^2^    = 3.842 x 0.2331  0.0025  = 358.23    N (Sp) = FP + TN  (1-P)  = 358.23 = 412  (1-0.13) |

3. NEWS2 score ≥5 in predicting critical care unit admission and in-hospital mortality for adult COVID-19 patients

Based on a study done by ([3](#_ENREF_3)), sensitivity and specificity for critical care unit admission and mortality prediction were 98% and 28% respectively.

With a prevalence of 5% of adult COVID patients in ED HCTM (P = 0.05), z = 1.96 (CI = 95%) and w = 0.05, sample sizes for sensitivity N(Sn) and specificity N(Sp) are calculated as below:

| Sensitivity N (Sn) | Specificity N (Sp) |
| --- | --- |
| TP + FN = z^2^ x Sn (1-Sn)  w^2^    = 1.96^2^ x 0.98 (1 - 0.98)  0.05^2^    = 3.842 x 0.0196  0.0025  = 30.12    N (Sn) = TP + FN  P  = 30.12 = 602  0.05 | FP + TN = z^2^ x Sp (1-Sp)  w^2^    = 1.96^2^ x 0.28 (1 – 0.28)  0.05^2^    = 3.842 x 0.2016  0.0025  = 309.82    N (Sp) = FP + TN  (1-P)  = 309.82 = 326  (1-0.05) |

| Outcome | Subgroup | N (Sn) | N (Sp) |
| --- | --- | --- | --- |
| Critical care unit admission | All Adult | 136 | 1362 |
|  | Sepsis | 1589 | 412 |
|  | COVID | 602 | 326 |
| In-hospital mortality | All Adult | 136 | 1362 |
|  | Sepsis | 1589 | 412 |
|  | COVID | 602 | 326 |

Conclusion: sample needed is 1589 subjects. With consideration of 20% missing data, the final sample size required for this study is 1589 + 20% = 1906.
