# Supplementary material for: National Early Warning Score 2 (NEWS2) as a prognostic tool for adult patients in emergency department: A retrospective observational study
Source: PLoS One. 2025 Jun 16;20(6):e0326058. doi: 10.1371/journal.pone.0326058 (PMC12169516; doi:10.1371/journal.pone.0326058)
Supplement: S2 Table — (DOCX) [file pone.0326058.s002.docx]

**SUPPORTING INFORMATION FILE 2**

**S1 Table: Baseline Characteristics of Sepsis Patients in ED Stratified by Hospital Admission, Critical Care Unit Admission and Mortality**

| **Baseline Characteristics** | **Patient Outcome** | | | | | | | | |
| --- | --- | --- | --- | --- | --- | --- | --- | --- | --- |
|  | **Hospital admission** | | | **Critical care unit admission** | | | **Mortality** | | |
|  | **No** | **Yes** | **P value** | **No** | **Yes** | **P value** | **No** | **Yes** | **P value** |
| **Total** n (%) | 2 (1.2) | 157 (98.7) |  | 138 (86.7) | 21 (13.2) |  | 133 (83.6) | 26 (16.3) |  |
| **Gender** n (%)  Male  Female | 2 (100)  0 (0) | 91 (58.0)  66 (42.0) | 0.511^c*^ | 78 (56.5)  60 (43.5) | 15 (71.4)  6 (28.6) | 0.197^a*^ | 76 (57.1)  57 (42.9) | 17 (65.4)  9 (34.6) | 0.435^a*^ |
| **Age** median (IQR) | 64 (54,74) | 67 (57,75) | 0.877^b*^ | 67 (57,75) | 68 (55,75) | 0.708^b*^ | 67 (57,75) | 66 (52,74) | 0.677^b*^ |
| **Vital signs** median (IQR)  HR  RR  sBP  dBP  T  SpO2 | 76 (64,89)  19 (18,20)  148 (129,168)  71 (66,77)  37.1 (37.3,37.0)  98 (99,97) | 102 (87,118)  22 (20,26)  128 (113,153)  75 (60,87)  37.6 (37.0,38.2)  95 (92,98) | 0.438^d*^  0.166^b*^  0.438^d*^  0.811^d*^  0.408^b*^  0.180^b*^ | 100 (87,115)  22 (20,25)  131 (116,155)  77 (62,88)  37.5 (37.0,38.2)  95 (92,98) | 116 (87,128)  26 (23,31)  110 (90,149)  58 (44,84)  38.0 (37.1,38.2)  92 (85,94) | 0.105^d*^  <0.001^b*^  0.034^d*^  0.015^d*^  0.371^b*^  <0.001^b*^ | 100 (88,116)  22 (20,26)  130 (116,156)  77 (62,89)  37.5 (37.0,38.2)  95 (92,98) | 114 (85,128)  24 (21,28)  123 (104,147)  66 (51,84)  37.6 (37.0,38.2)  94 (96,87) | 0.179^d*^  0.027^b*^  0.133^d*^  0.096^d*^  0.926^b*^  0.108^b*^ |
| **Supplemental oxygen** n (%) | 0 (0) | 98 (62.4) | 0.146^c*^ | 81 (58.7) | 17 (81.0) | 0.057^a*^ | 78 (58.6) | 20 (76.9) | 0.080^a*^ |
| **State of consciousness** n (%) | 2 (100) | 122 (77.7) | 1.000^c*^ | 111 (80.4) | 13 (61.9) | 0.056^a*^ | 108 (81.2) | 16 (61.5) | 0.027^a*^ |
| **Comorbidities** n (%)  Hypertension  Diabetes  CVD  COPD  Renal disease  CVA  Malignancy | 2 (100)  1 (50)  1 (50)  0 (0)  2 (100)  0 (0)  0 (0) | 105 (66.9)  72 (45.9)  53 (33.8)  6 (3.8)  31 (19.7)  18 (11.5)  28 (17.8) | 1.000^c*^  1.000^c*^  1.000^c*^  1.000^c*^  0.042^c*^  1.000^c*^  1.000^c*^ | 92 (66.7)  64 (46.4)  46 (33.3)  4 (2.9)  29 (21.0)  18 (13.0)  26 (18.8) | 15 (71.4)  9 (42.9)  8 (38.1)  2 (9.5)  4 (19.0)  21 (100)  2 (9.5) | 0.665^a*^  0.763^a*^  0.668^a*^  0.180^c*^  1.000^c*^  0.133^a*^  0.374^c*^ | 87 (65.4)  61 (45.9)  43 (32.3)  5 (3.8)  27 (20.3)  16 (12.0)  21 (15.8) | 20 (76.9)  12 (46.2)  11 (42.3)  1 (3.8)  6 (23.1)  2 (7.7)  7 (26.9) | 0.435^a*^  0.978^a*^  0.326^a*^  1.000^c*^  0.750^a*^  0.740^c*^  0.173^a*^ |
| **Diagnosis** n (%)  Medical illness  Surgical disease  Infectious illness  Traumatic illness | 0 (0)  0 (0)  2 (100)  0 (0) | 84 had simultaneous diagnosis  64 (40.7)  19 (12.1)  157 (100)  1 (0.6) | 1.000^c*^ | 75 had simultaneous diagnosis  56 (40.5)  18 (13.0)  138 (100)  1 (0.7) | 9 had simultaneous diagnosis  8 (38.0)  1 (4.7)  21 (100)  0 (0) | 1.000^c*^ | 74 had simultaneous diagnosis  56 (42.1)  17 (12.7)  133 (100)  1 (0.7) | 10 had simultaneous diagnosis  8 (30.7)  2 (7.6)  26 (100)  0 (0) | 1.000^c*^ |
| **NEWS2** median (Q1,Q3) | 0 (0,0) | 7 (4,9) | 0.022^b*^ | 6 (3,9) | 10 (7,13) | <0.001^b*^ | 6 (4,9) | 8 (7,12) | 0.010^b*^ |

*A 2-sided *P* value of < 0.05 indicate statistical significance

a Pearson Chi-square test

b Mann-Whitney U test

c Fisher’s exact test

d Student’s t test

**S2 Table: Baseline Characteristics of COVID-19 Patients in ED Stratified by Hospital Admission, Critical Care Unit Admission and Mortality**

| **Baseline Characteristics** | **Patient Outcome** | | | | | | | | |
| --- | --- | --- | --- | --- | --- | --- | --- | --- | --- |
|  | **Hospital admission** | | | **Critical care unit admission** | | | **Mortality** | | |
|  | **No** | **Yes** | **P value** | **No** | **Yes** | **P value** | **No** | **Yes** | **P value** |
| **Total** n (%) | 11 (27.5) | 29 (72.5) |  | 37 (92.5) | 3 (7.5) |  | 38 (95.0) | 2 (5.0) |  |
| **Gender** n (%)  Male  Female | 6 (54.5)  5 (45.5) | 16 (55.2)  13 (44.8) | 1.000^c*^ | 21 (56.8)  16 (43.2) | 1 (33.3)  2 (66.7) | 0.579^c*^ | 20 (52.6)  18 (47.4) | 2 (100)  0 (0) | 0.492^c*^ |
| **Age** median (IQR) | 72 (28,87) | 75 (70,79) | 0.671^b*^ | 74 (66,81) | 77 (69,79) | 0.777^b*^ | 74 (66,80) | 77 (5.6) | 0.709^b*^ |
| **Vital signs** median (IQR)  HR  RR  sBP  dBP  T  SpO2 | 97 (77,122)  20 (19,20)  153 (108,156)  78 (68,90)  37.5 (37.0,38.5)  97 (96,98) | 96 (78,109)  20 (18,24)  135 (116,154)  80 (62,91)  38.0 (37.2,38.6)  97 (90,98) | 0.535^d*^  0.182^b*^  0.716^b*^  0.624^d*^  0.215^d*^  0.490^b*^ | 94 (77,109)  20 (18,21)  139 (119,154)  80 (63,90)  38.0 (37.1,38.6)  97 (98,94) | 127 (96,-)  28 (26,36)  110 (82,184)  68 (43,91)  38.2 (38.0,-)  88 (80,92) | 0.069^d*^  0.006^b*^  0.555^b*^  0.265^d*^  0.328^b*^  0.022^b*^ | 95 (110,77)  20 (24,18)  138 (154,114)  77 (90,62)  38.0 (38.5,37.1)  97 (98,93) | 105 (-,97)  20 (20,20)  136 (-,117)  98 (-, 98)  38.1 (-,37.6)  90 (-,82) | 0.564^d^*  0.898^b*^  0.991^d*^  0.081^d*^  0.748^d*^  0.660^b*^ |
| **Supplemental oxygen** n (%) | 0 (0) | 15 (51.7) | 0.003^c*^ | 12 (32.4) | 3 (100) | 0.046^c*^ | 14 (36.8) | 1 (50) | 1.000^c*^ |
| **State of consciousness** n (%) | 11 (100) | 26 (89.6) | 0.548^a*^ | 35 (94.5) | 2 (66.6) | 0.214^c*^ | 35 (92.1) | 2 (100) | 1.000^c*^ |
| **Comorbidities** n (%)  Hypertension  Diabetes  CVD  COPD  Renal disease  CVA  Malignancy | 4 (36.4)  0 (0)  0 (0)  1 (9.1)  0 (0)  0 (0)  1 (9.1) | 22 (75.9)  16 (55.2)  10 (34.5)  0 (0)  6 (20.7)  7 (24.1)  1 (3.4) | 0.029^c*^  0.001^c*^  0.038^c*^  0.275^c*^  0.162^c*^  0.159^c*^  0.479^c*^ | 24 (64.9)  14 (37.8)  10 (27.0)  1 (2.7)  6 (16.2)  7 (18.9)  2 (5.4) | 2 (66.6)  2 (66.6)  0 (0)  0 (0)  0 (0)  0 (0)  0 (0) | 1.000^c*^  0.553^c*^  0.560^c*^  1.000^c*^  1.000^c*^  1.000^c*^  1.000^c*^ | 24 (63.2)  14 (36.8)  10 (26.3)  1 (2.6)  5 (13.2)  6 (15.8)  2 (5.3) | 2 (100)  2 (100)  0 (0)  0 (0)  1 (50)  1 (50)  0 (0) | 0.533^c*^  0.154^c*^  1.000^c*^  1.000^c*^  0.281^c*^  0.323^c*^  1.000^c*^ |
| **Diagnosis** n (%)  Medical illness  Surgical disease  Infectious illness  Traumatic illness | 1 had simultaneous diagnosis  0 (0)  0 (0)  11 (100)  1 (9.0) | 11 had simultaneous diagnosis  10 (34.4)  0 (0)  29 (100)  1 (3.4) | 0.250^c*^ | 10 had simultaneous diagnosis  8 (21.6)  0 (0)  37 (100)  2 (5.4) | 2 had simultaneous diagnosis  2 (66.6)  0 (0)  3 (100)  0 (0) | 1.000^c*^ | 11 had simultaneous diagnosis  10 (26.3)  0 (0)  38 (100)  1 (2.6) | 1 had simultaneous diagnosis  0 (0)  0 (0)  2 (100)  1 (50) | 0.250^c*^ |
| **NEWS2** median (Q3,Q1) | 2 (1,3) | 4 (1,9) | 0.067^b*^ | 3 (1,5) | 11 (10,17) | 0.008^b*^ | 3 (1,5) | 4.5 (-,2) | 0.662^b*^ |

*A 2-sided *P* value of < 0.05 indicate statistical significance

a Pearson Chi-square test

b Mann-Whitney U test

c Fisher’s exact test

d Student’s t test

**References**

1. Thoren A, Joelsson-Alm E, Spangfors M, Rawshani A, Kahan T, Engdahl J, et al. The predictive power of the National Early Warning Score (NEWS) 2, as compared to NEWS, among patients assessed by a Rapid response team: A prospective multi-centre trial. Resusc Plus. 2022;9:100191.

2. Mellhammar L, Linder A, Tverring J, Christensson B, Boyd JH, Sendi P, et al. NEWS2 is superior to qSOFA in detecting sepsis with organ dysfunction in the emergency department. Journal of clinical medicine. 2019;8(8):1128.

3. Baker KF, Hanrath AT, Schim van der Loeff I, Kay LJ, Back J, Duncan CJ. National Early Warning Score 2 (NEWS2) to identify inpatient COVID-19 deterioration: a retrospective analysis. Clin Med (Lond). 2021;21(2):84-9.
